# Relative age effects in Rugby union and Rugby league: a systematic review and meta-analysis

**DOI:** 10.3389/fspor.2026.1809947

**Published:** 2026-06-05

**Authors:** P. R. Brustio, S. Cobley, A. N. Ungureanu, A. B. T. McAuley, C. Lupo, G. Boccia, K. Till, A. L. Kelly

**Affiliations:** 1 Department of Clinical and Biological Sciences, University of Turin, Turin, Italy; 2 Neuromuscular Function Research Group, School of Exercise & Sport Sciences, University of Turin, Turin, Italy; 3 Discipline of Exercise & Sport Science, Faculty of Health Sciences, The University of Sydney, Sydney, NSW, Australia; 4 Department of Life Sciences and Systems Biology, University of Turin, Turin, Italy; 5 Research for Athlete and Youth Sport Development (RAYSD) Lab, Centre for Life and Sport Sciences (CLaSS), Faculty of Health, Education and Life Sciences, Birmingham City University, Birmingham, United Kingdom; 6 Department of Medical Sciences, University of Turin, Turin, Italy; 7 Carnegie School of Sport, Leeds Beckett University, Leeds, United Kingdom

**Keywords:** competition level, RAE, relative age, selection bias, sex differences, talent identification, youth sport

## Abstract

**Introduction:**

Relative Age Effects (RAEs) are prominent factors affecting youth sport participation and talent identification and selection processes. In rugby, RAEs appear to be prevalent in both Rugby League (RL) and Rugby Union (RU), yet no study has quantitatively synthesised the available evidence across codes. This systematic review and meta-analysis aimed to identify RAE prevalence, magnitude, and moderating factors (i.e., sex, age, and competition level) across rugby codes, using the extreme-quartile contrast between Q1 and Q4.

**Methods:**

A systematic search in CINAHL, Ovid, PsycINFO, PubMed, Scopus, and Web of Science identified 26 eligible studies. Distribution data were synthesised using odds ratio meta-analyses with an inverse-variance random-effects model and separate analyses by sex, age, and competition level.

**Results:**

Male players showed a significant overrepresentation in Q1 vs. Q4 (34.8% vs. 16.9%; OR = 1.99), with smaller but similar effects in female players (27.5% vs. 23.6%; OR = 1.15). In males, RAEs were stronger in youth than senior categories (OR = 2.42 vs. 1.41), and in younger (U7–U14; U15–U17) vs. older age groups (U18-U20).

**Discussion:**

Descriptively, RAEs appeared larger at higher competition levels, particularly in youth males (regional/Local: OR = 2.14; national: OR = 2.76). Overall, findings demonstrate that RAEs are pervasive in rugby and persist across sex, age, and competition level. However, the magnitude of the pooled estimates should be interpreted cautiously in light of the substantial between-study heterogeneity and the attenuation of the male effect following adjustment.

## 1 Introduction

Most sports governing bodies and federations consider talent identification and development pivotal for their respective sports' growth and success (1–3). These programs are designed and delivered to systematically nurture young athletes, guiding them from the initial stages of participation to the attainment of high performance. However, identifying a young, talented athlete is often complex due to the numerous factors affecting an athlete's development (3, 4). These direct factors, such as genetic makeup, technical and tactical skills, along with indirect factors, such as sociocultural influences and organizational structures, collectively shape the opportunities to be identified and selected.

Among these indirect factors, arguably one of the most studied phenomena, is the Relative Age Effect (RAEs) (5). RAEs reflect the (dis)advantages generated by the interaction between chronological age and the commonly used (bi)annual age cut-off criteria for grouping youth players by developmental stage, impacting the identification, selection, and development processes (6). Being born close to the start of an annual age-cut-off date (i.e., relatively older) often provides a range of anthropometric, physiological, and psychosocial development benefits. These age cut-offs are commonly associated with generic performance advantages in many youth sports settings relative to those born in the last three months (i.e., relatively younger) of the same annual cohort (7–9). Consequently, relatively older sporting participants are more frequently, consciously or unconsciously, favoured by talent scouts, coaches, and other stakeholders at the youth level leading to their disproportionate over-representation.

In rugby, RAEs are prevalent in both Rugby League (RL) and Rugby Union (RU) [2,7]. Being relatively older likely leads to performance advantages in crucial rugby actions, such as rucking, running with the ball, scrummaging, and tackling (10–12). Further, the high physical demands of RL and RU may make rugby more vulnerable to RAEs (6, 13–16). Indeed, several factors likely affect RAE magnitudes in rugby, including (a) sex, (b) age, (c) competition level, (d) sociocultural context, and (e) playing position (17).

Regarding sex, RAE magnitudes were found to be larger in males than in females (18, 19). For example, Till et al. (18) evaluated RAEs in male and female English age-grade rugby league (from U7 to senior level), revealing a consistent advantage for Q1 (i.e., those born in the first birth quarter of the selection year) players in male youth and senior rugby league compared to Q4 (i.e., those born in the fourth birth quarter of the selection year). However, few RAEs were present in the female sample. In the same national context focused on RU, Kelly et al. (19) identified RAEs in all male annual age categories and nine of 12 female annual age categories, highlighting how sex plays an important role in the prevalence of RAEs.

Related to age and competition level, RAEs have been more pronounced during childhood and adolescence, with effect sizes decreasing as age increases (2, 7, 20). For instance, in their examination of Welsh rugby league, Lewis et al. (21) identified that at grassroots level from U7 to U19, Q1 participation percentages were, on average, higher (29%) than Q4 participation percentages (21%). However, with competition level, relative age magnitude increased, whereby in Welsh competition (U16 categories), the odds of being selected for Q1 vs. Q4 players (i.e., odds ratios) were approximately 2.7, increasing to 4.7 at the regional level, and reaching ∼12 at the national level (21).

Despite RAEs seeming almost ever-present in rugby codes at the youth level, whether RAEs remain prevalent at the senior level are unclear and may be dependent on socio-cultural context (16). While some research indicates residual “knock-on effect” may occur, where Q1 overrepresentation continues into senior levels (14, 16), other studies suggest either RAEs dissipate with age (13, 22, 23) or even reverse (i.e., Q4 overrepresentation) (24–26). For example, RAEs were absent in England where the proportion of players were about 25% in each quartile for senior professional players [Kelly, Till, et al. (23),]. Similarly, studies from France have indicated weak or non-existent RAEs (13, 27). In contrast, data from the Italian RU context indicated that players born at the beginning of the selection cut-off period were approximately 1.3 times more likely to be represented than those born at the end (14).

Mixed results also emerged when considering players position. For example, a study from France indicated that forward players exhibited RAEs, whereas backs did not (27). On the contrary, at international level, data suggested a skewed distribution for backs (favouring Q1) but reversal for forwards (favouring Q4) (28). Brustio et al. (16) also suggested that whilst RAEs were weak, they persisted in backs and forwards but were inconsistent for scrum-halves. These data suggest that the sociocultural context or rugby system may lead to or reduce RAE occurrence of no RAEs, or relative age reversal.

Although a narrative review has summarized the current state of RAEs in Rugby codes via a qualitative synthesis (11), to our knowledge, no study has conducted a systematic quantitative synthesis. This is particularly important, as this is a growing field of research influenced by several factors (i.e., sex, age, competition level, sociocultural context, and playing position) that require greater clarity. By doing so may help positively impact the effectiveness, efficiency, ethics of talent development pathways in RL and RU by providing recommendations based on the overall literature. Therefore, this systematic review and meta-analysis aimed to consolidate the current knowledge on RAEs in RU and RL by identifying their prevalence, magnitude, and moderating factors (i.e., sex, age, nation, and competition level) across rugby codes, using the extreme-quartile comparison between Q1 and Q4.

## 2 Methodology

Relative age studies conducted within rugby were identified through a systematic search of six electronic databases, including CINAHL, OVID, PSYCINFO, PUBMED, Scopus, and Web of Science. Searches were performed using the following strings: (“relative age effect” OR “relative age effects” OR “RAE” OR “RAEs” OR “relative age” OR “age effect” OR “birthdate”) AND (“rugby” OR “rugby union” OR “rugby league”). The search included articles published until March 2025. In addition, the references of the included articles were screened to identify potentially eligible studies.

### 2.1 Inclusion and exclusion criteria

Studies were included if they: (1) reported data on RAEs in the context of RU and/or RL context both in males and/or females; (2) assessed any age range until aged 50 years; (3) analysed any competition level; and, (4) were published in English language. Studies were excluded if they: (1) did not provide the absolute or relative distribution of birth dates; (2) included participants who were not rugby players; (3) examined RAEs in a school sport or physical education context; (4) summed sub-samples from different sports; (5) analysed master players (i.e., up to 50 years and retaining competitive amateur/professional status); and, (6) were sources of reviews, meta-analyses, abstracts, books, statements, letters, editorials, grey data and unpublished data, or commentaries (although these were screened to identify potentially eligible studies).

### 2.2 Systematic search

**Figure 1:**
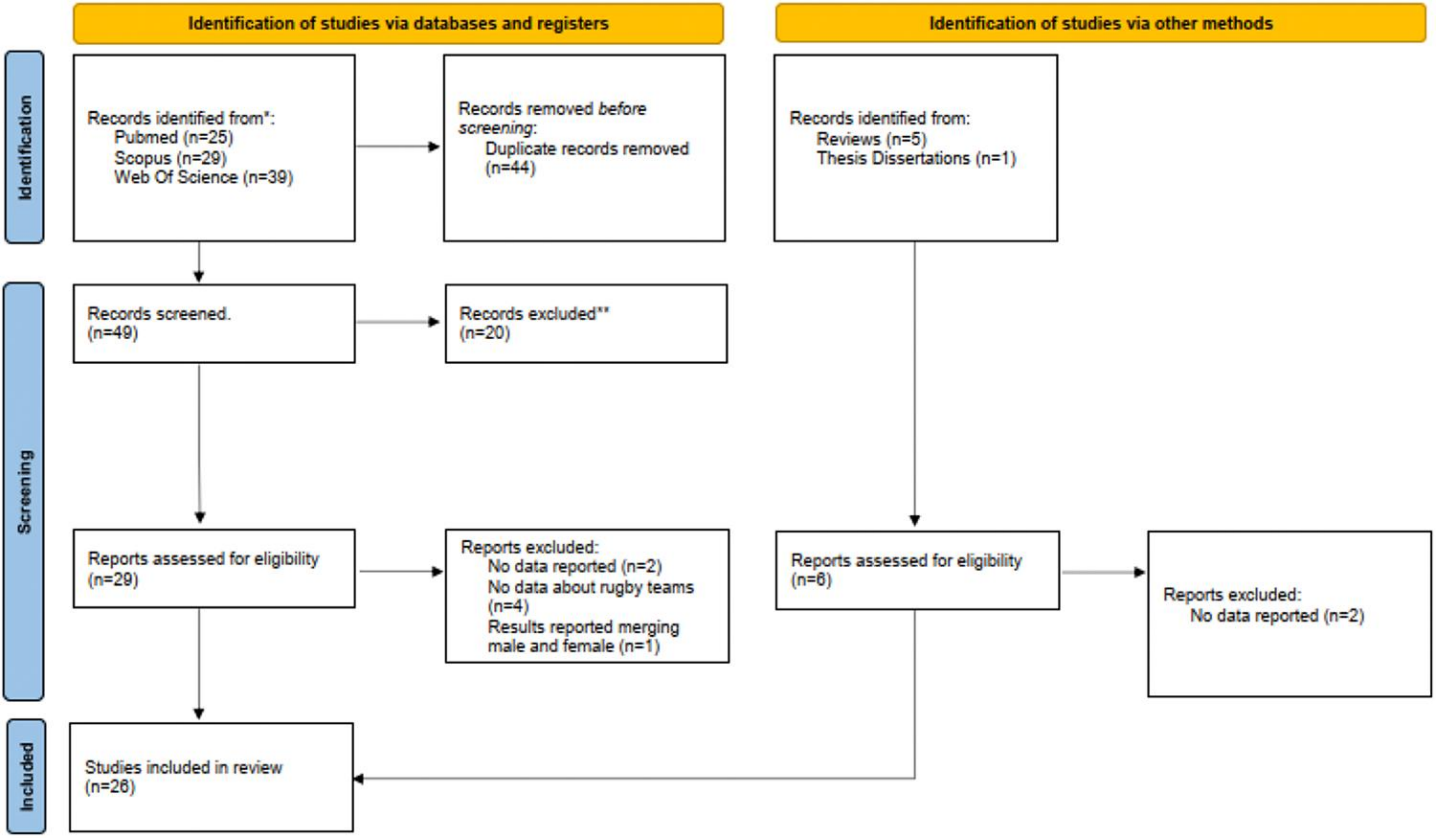
Flow diagram for screening and selection of studies.

A summary of the study screening protocol and selection process is shown in Figure 1. Following the search, the first author (P.R.B) removed duplicates. The selection process was performed by two authors (P.R.B. and A.N.U.) who independently screened study titles and/or abstracts to identify those potentially meeting inclusion criteria. Full texts of potentially eligible studies were then assessed for eligibility. Disagreements were resolved by discussion with a third author (A.L.K). If any potentially informative data was missing, respective corresponding authors of studies were contacted. Studies without responses or data available were excluded from the analysis.

**Figure 2:**
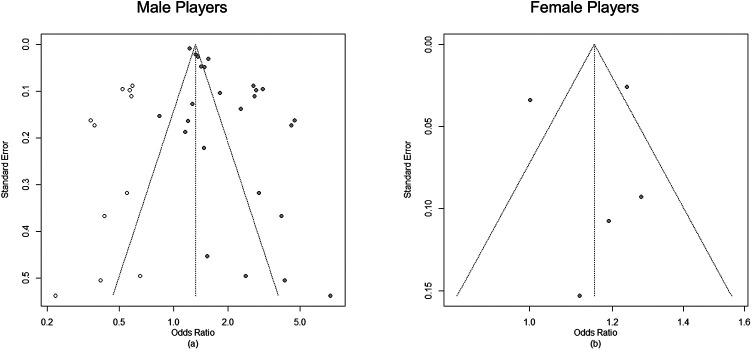
Funnel plot of standard error by log odds ratio (Q1 vs. Q4) for male **(a)** and female **(b)** sample. Observed studies are shown as grey circle while the imputed studies of Trim-and-Fill method are shown as open circles.

### 2.3 Study quality assessment

Following similar meta-analysis procedures as Smith et al. (2) who examined RAEs across female sport, an adapted version of the Strengthening the Reporting of Observational Studies in Epidemiology checklist was used to assess study quality. The checklist included 14 items grouped into five categories: (1) Abstract (#1 item); (2) Introduction (#2–#3 items); (3) Methods (#4–#7 items); (4) Results (#8–#10 items); and, (5) Discussion (#11–#14 items). Items #5, #7, and #10 contain further sub-items. Each item was scored as 1 (i.e., present) or 0 (i.e., not present). A score of 0 was assigned for “no or insufficient information provided” and 1 for “item is explicitly described”. An overall score of 5–9 was considered “low quality”, 10–11 “medium quality”, and 12–14 “high quality” (2). Two independent authors (A.N.U. and C.L.) completed study quality assessment.

### 2.4 Meta-analyses

A Microsoft Excel® (Microsoft Corp., Redmond, WA, USA) spreadsheet was compiled, collating study information (i.e., lead author and year of publication) and five study characteristics (i.e., Sex Rugby Type, Nation, Age Categories, and Competition Level). Corresponding relative age data were then extracted from each manuscript.

For each study, the OR estimate was calculated using the following formula:

$$OR=\frac{nQ1_{obs}/nQ1_{exp}}{nQ4_{obs}/nQ4_{exp}}$$

Here, $nQ1_{\mathrm{obs}}$ and $nQ4_{\mathrm{obs}}$ represent the observed frequency of players in Q1 and Q4, and $nQ1_{\exp}$ and $nQ4_{\exp}$ represent the expected frequency of players in Q1 and Q4. Log odds ratios (logOR) and their standard errors (seTE) derived from the observed and expected values were calculated. The expected frequency was calculated by assuming equal distributions among the samples (i.e., 25% in each quartile). This approach was adopted according to previous meta-analytic work on the topic (2, 7, 20) as a pragmatic strategy to allow comparable effect-size estimation across studies conducted in different countries, years, and competitive contexts. The primary meta-analytic comparison was defined *a priori* as Q1 vs. Q4. This extreme-quartile contrast was selected to provide a consistent and directly interpretable effect-size metric according to studies on RAE. Summary statistics, including the pooled OR and 95% confidence interval (95% CI), were calculated to provide effect sizes across studies. Overall summary estimates were calculated using an invariance random effects model (29). Heterogeneity was evaluated using the *I^2^* index. The *I^2^*statistic was used to quantify the proportion of observed variance attributable to differences in true effect sizes, rather than sampling error. The level of heterogeneity in the *I^2^* index was interpreted as low (25 to ≤50%), moderate (50 to ≤75%), and large (>75%) (30). Overall, OR magnitudes were determined using the following thresholds: small: OR ≥ 1.50; medium: OR ≥ 2.50; large: OR ≥ 4.30 (20). To determine the potential presence of publication bias, funnel plot asymmetry for accumulated male and female samples was initially assessed by plotting Log OR estimates against corresponding standard error. To confirm asymmetry, the Egger test was conducted (31), with follow-up Trim-and-Fill (32) procedures applied if asymmetry was identified. To determine the influence of potential moderating factors based on data available, follow-up sub-group analyses were conducted. Given the substantial imbalance in several moderator categories and the presence of multiple non-independent effect sizes derived from the same studies, meta-regression was not performed and subgroup analyses were used as a more conservative exploratory strategy (for details see the following section). All statistical analyses were conducted using the packages “meta” with the “metagen” function (33) of R (Version 4.0.0; R Core Team, Foundation for Statistical Computing, Vienna, Austria).

### 2.5 Subgroup analyses

Different subgroup analyses were conducted to determine whether age and competition level moderated RAEs. A primary analysis categorized studies into two groups based on player age: Youth (aged 7–20 years) and Senior (over aged 20 years). When studies reported different age categories (e.g., a mix of youth and senior samples), data were merged to align with the predefined age groups. Subsequently, an additional sub-analysis was conducted on studies that exclusively included youth players. To create more homogeneous age groups, and because not all studies reported data separately for each individual year, the samples were categorized into three groups: U7–U14 (aged 7–14 years), U15–U17 (aged 15–17 years), and U18–U20 (aged 18–20 years). When studies reported different age categories (e.g., U12, U13, U14), data were aggregated to align with the designated age classifications.

Further sub-analysis investigated the potential influence of competition level, considering youth and senior samples separately. Youth samples were classified as either local/regional or national level, whereas senior samples were categorized as national or international level. Due to the limited number of studies including female participants (*n* = 5), only the Youth vs. Senior sub-analysis was performed.

## 3 Results

### 3.1 Studies systematically identiﬁed

Figure 1 summarises the systematic search process steps and number of articles identified at each stage. Initial database searches identified 49 studies, and six studies were identified through other sources (i.e., reviews on the topic and thesis dissertations). Article titles and abstracts were then screened. After further removal of studies which did not provide RAE data, 26 studies met the inclusion and reporting criteria for the final analysis.

### 3.2 Study characteristics

Table 1 summarises the characteristics of identified studies in male and female context according to the following items according to age category (i.e., Youth vs. Senior): (1) general information (i.e., authors and years of publication, type of rugby competition, nation); and, (2) relative age results (i.e., number of subjects analysed, percentage of players in different birth quartiles, estimate OR, and 95%CI).

**Table 1:** Studies characteristic, quartile distributions and OR between Q1 and Q4 according to the gender and the age category.

**Authors**	**Type**	**Nation**	**Youth Vs. Senior**	**N**	**Q1%**	**Q2%**	**Q3%**	**Q4%**	**OR [95%CI]**
Male Players									
Brustio et al. (26, 54)	Rugby Union	International*	Senior	7,143	28.8	23.1	27.8	20.3	1.42 [1.29, 1.56]
Cobley et al., (15)	Rugby League	New Zealand/Australia	Senior	1,509	33.3	25.7	22.6	18.4	1.81 [1.47, 2.22]
Cobley et al. (24)	Rugby League	UK	Youth	23,943	29.6	25.0	23.7	21.7	1.36 [1.29, 1.43]
Delorme et al. (13)	Rugby Union	France	Senior	346	27.2	30.1	24.3	18.5	1.47 [0.95, 2.27]
Dimundo (a) et al., 2021	Rugby Union	England	Youth	74	33.8	32.4	20.3	13.5	2.50 [0.95, 6.60]
Dimundo (b) et al., 2021	Rugby Union	England	Youth	78	42.3	26.9	20.5	10.3	4.12 [1.54, 11.14]
Fernley et al. (39)	Rugby Union	Australia	Youth	1,895	37.6	29.4	19.9	13.1	2.86 [2.36, 3.46]
Jones et al. (28)	Rugby Union	International**	Senior	691	22.0	26.6	25.0	26.4	0.84 [0.62, 1.13]
Kearney (a) et al. (22)	Rugby Union	France	Senior	1,991	39.9	26.7	20.6	12.8	3.11 [2.59, 3.77]
Kearney (b) et al. (27)	Rugby Union	International***	Senior	6,679	30.7	25.4	23.2	20.7	1.48 [1.35, 1.63]
Kelly et al., (11, 23)	Rugby Union	England	Youth	1,963	39.9	27.7	20.4	12.0	3.32 [2.74, 4.02]
			Senior	329	27.1	21.3	27.4	24.3	1.11 [0.72, 1.71]
Kelly et al., (19, 50)	Rugby Union	England	Youth	216,256	28.0	24.8	24.4	22.8	1.23 [1.21, 1.25]
Kelly et al., (38)	Rugby Union	England	Youth	787	30.2	24.7	22.4	22.7	1.33 [1.01, 1.75]
			Senior	189	21.7	28.6	28.0	21.7	1.00 [0.55, 1.81]
Lewis et al., (21)	Rugby Union	Welsh	Youth	32,485	29.0	25.7	23.4	21.9	1.32 [1.27, 1.38]
Lupo et al. (14)	Rugby Union	Italy	Senior	572	29.5	25.2	20.6	24.7	1.20 [0.87, 1.65]
McCarthy et al. (40)	Rugby Union	England	Youth	118	41.5	31.4	18.6	8.5	4.90 [2.1, 11.45]
			Senior	36	27.8	30.6	27.8	13.9	2.00 [0.49, 8.24]
McCarthy et al. (41)	Rugby Union	England	Youth	821	41.0	22.0	21.0	16.0	2.57 [1.95, 3.40]
			Senior	45	11.1	17.8	40.0	31.1	0.36 [0.10, 1.34]
Morley et al. (42)	Rugby League	England	Youth	84	27.4	21.4	33.3	17.9	1.53 [0.63, 3.73]
Nakata et al. (43)	Rugby Union	Japan	Senior	479	24.2	29.6	25.3	20.9	1.16 [0.80, 1.68]
Owen et al. (34)	Rugby Union	England	Youth	1,424	39.6	26.3	19.9	14.2	2.79 [2.25, 3.48]
Roberts et al. (44)	Rugby Union	England	Youth	168	44.0	21.4	19.6	14.9	2.96 [1.59, 5.55]
Till (a) et al. (12)	Rugby League	England	Youth	767	45.8	24.9	19.6	9.8	4.68 [3.39, 6.42]
Till (b) et al. (18)	Rugby League	England	Youth	16,432	31.2	25.9	22.6	20.3	1.54 [1.45, 1.63]
			Senior	628	34.4	23.2	24.5	17.8	1.93 [1.40, 2.65]
Till et al. (45)	Rugby League	England	Youth	81	54.3	27.2	11.1	7.4	7.33 [2.55, 21.00]
Till et al. (46)	Rugby League	England	Youth	650	47.4	23.5	18.5	10.6	4.46 [3.19, 6.29]
Female Players									
Cobley et al. (24)	Rugby League	England	Youth	1,809	29.9	19.9	26.9	23.4	1.28 [1.06, 1.53]
Kelly et al., (19, 50)	Rugby Union	England	Youth	23,569	28.1	24.8	24.4	22.7	1.24 [1.18, 1.30]
Kelly et al., (35)	Rugby Union	UK	Youth	371	30.2	22.6	26.4	20.8	1.45 [0.97, 2.19]
			Senior	1,004	26.9	24.4	24.4	24.3	1.11 [0.86, 1.42]
Lemez et al. (47)	Rugby Union	Canada	Youth	11,691	23.9	25.0	26.5	24.6	0.97 [0.90, 1.04]
		New Zealand	Youth	1,785	27.2	25.2	26.7	21.0	1.30 [1.07, 1.57]
		International	Senior	498	23.1	24.9	25.3	26.7	0.86 [0.61, 1.23]
Till (b) et al. (18)	Rugby League	UK	Youth	409	27.9	21.5	26.9	23.7	1.18 [0.80, 1.73]
			Senior	261	26.4	25.7	22.2	25.7	1.03 [0.64, 1.67]

A total of 318,663 male and 41,379 female rugby players were included in the meta-analysis. Overall, 21 studies focused only on males, two only on females, and three on both sexes. Most studies focused on RU (74.1%) and on male players (83.4%). Eight studies (30.7%) were published in the last four years (16, 19, 23, 34–38), while 18 (69.3%) were published between 2009 and 2019 (6, 12–15, 18, 22, 27, 28, 39–47).

The majority of studies (65.4%) included a UK sample (6, 12, 18, 19, 21, 23, 34–38, 40–42, 44–46), followed by Oceania (Australia, *n* = 2; New Zealand, *n* = 2) (15, 39, 47), Europe (France, *n* = 2; Italy, *n* = 1) (13, 14, 27), North America (Canada, *n* = 1) (47), and Asia (Japan, *n* = 1) (43). Four studies (16, 22, 28, 47) included an international sample representing the national RU teams of recent years (e.g., from World Rugby Ranking).

Twelve articles (6, 12, 21, 23, 34, 36, 37, 39, 42, 44–46) published data only on youth players (aged 7–21 years), whereas eight only analysed senior players (13–16, 22, 27, 28, 43), and seven considered both senior and youth players (11, 18, 35, 38, 40, 41, 47).

#### 3.2.1 Male study characteristics: age and competition level

Considering studies on males that focused on youth players (*n* = 17), different sub-age categories were examined. Nine (33.3%) reported data on U8–U14 (three on RU and six on RL) (6, 12, 18, 19, 21, 42, 44–46), eleven (40.7%) reported data on U15–U17 (6, 11, 18, 19, 21, 34, 36–39, 44), and seven (26%) reported data on U17–U20 (6, 18, 19, 21, 23, 36, 38).

In the Youth category, ten (58.8%) reported data at the Local/Regional level (6, 12, 18, 19, 21, 23, 34, 38, 45, 46), and seven (41.2%) at the National level (18, 36, 37, 39, 42, 44, 46). In the Senior category, data from ten studies (75%) were reported at the National level (11, 13–15, 18, 27, 38, 40, 41, 43), and five at the International level (25%) (16, 18, 22, 23, 28).

#### 3.2.2 Female study characteristics

Considering female players, studies examining on youth players (*n* = 6), five (83.3%) reported data on U8–U14, with two on RU and two on RL (6, 18, 19, 35, 47). One study (16.7%) reported data on U15–U17 players (35), and three studies reported data on Senior players (18, 35). Regarding competition level, three studies reported data on the Regional/Local level (6, 18, 19), five on the National level (35, 47), and one on the International level (47).

### 3.3 Study quality

According to the selected criteria in males, 13 of 24 studies (54.2%) were considered “high quality”, 22 (45.8%) were considered “medium quality”, and none were considered “low quality”. In females, all studies were considered of “high quality”. Criteria commonly absent in reporting related to explaining and reporting the reference baseline distribution as well as reporting statistical estimate(s) and precision (e.g., 95% CI). Additional information about the quality scores is presented in Table 2 for male and female players.

**Table 2:** Study quality of selected articles.

Authors	Abstract	Introduction		Methods							Results				Discussion				Scored
	#1	#2	#3	#4	#5			#6	#7		#8	#9	#10		#11	#12	#13	#14	
					a	b	c		a	b			a	b					
Male																			
Brustio et al. (26, 54)	1	1	1	1	1	1	1	1	1	1	1	1	1	1	1	1	1	1	14
Cobley et al. (15	1	1	1	1	1	1	1	0	1	0	1	1	0	1	1	1	1	1	12
Cobley et al. (24)	1	1	1	1	1	1	1	1	1	1	1	1	1	1	1	0	0	1	12
Delorme et al. (13)	1	1	1	1	0	1	1	1	1	1	1	0	1	1	1	1	1	1	11
Dimundo (a) et al. 2021	1	1	1	0	0	1	1	0	1	1	1	1	1	1	1	1	1	1	11
Dimundo (b) et al. 2021	1	1	1	0	0	1	1	0	1	1	1	1	1	1	1	1	1	1	11
Fernley et al. (39)	1	1	1	1	1	1	1	0	1	1	1	1	1	1	1	1	1	1	13
Jones et al. (28)	1	1	1	1	1	1	1	0	1	1	1	0	1	1	1	0	1	1	11
Kearney (a) et al. (22)	1	1	1	1	1	1	1	1	1	1	1	0	1	1	1	0	0	0	10
Kearney (b) et al. (27)	1	1	1	0	1	1	1	1	1	1	1	0	1	1	1	1	1	1	12
Kelly et al. (11, 23)	1	1	1	1	1	1	1	1	1	1	1	1	1	1	1	1	1	1	14
Kelly et al. (19, 50)	1	1	1	1	1	1	1	0	1	1	1	1	1	1	1	1	1	1	13
Kelly et al. (38)	1	1	1	1	1	1	0	1	1	0	1	1	1	0	1	1	1	1	12
Lewis et al. (21)	1	1	1	1	1	1	1	1	1	1	1	1	0	1	1	1	1	1	13
Lupo et al. (14)	1	1	1	1	1	1	1	0	1	1	0	1	1	0	1	1	1	1	11
McCarthy et al. (40)	1	1	1	1	1	1	1	1	1	1	1	0	0	0	1	0	1	1	11
McCarthy et al. (41)	1	1	1	1	1	1	1	1	1	1	1	0	0	1	1	0	1	1	11
Nakata et al. (43)	1	1	1	1	1	1	1	1	1	1	1	0	0	0	1	1	1	1	12
Owen et al. (34)	1	1	1	1	1	1	1	0	1	0	1	0	1	0	1	1	1	1	10
Roberts et al. (44)	1	1	1	1	1	1	1	0	1	1	1	1	1	1	1	1	1	1	12
Till (a) et al. (12)	1	1	1	1	1	1	1	0	1	1	1	1	1	1	1	0	1	1	12
Till (b) et al. (18)	1	1	1	1	1	1	1	1	1	1	1	1	0	0	1	1	1	1	14
Till et al. (45)	1	1	1	1	0	1	1	0	1	1	1	0	0	0	1	1	1	1	10
Till et al. (46)	1	1	1	1	1	1	0	0	1	1	1	0	1	1	1	1	1	1	10
Female																			
Cobley et al. (24)	1	1	1	1	1	1	1	1	1	1	1	1	1	1	1	0	0	1	12
Kelly et al., (19, 50)	1	1	1	1	1	1	1	0	1	1	1	1	1	1	1	1	1	1	13
Kelly et al., (35)	1	1	1	1	1	1	1	1	1	1	1	1	1	1	1	1	1	1	14
Lemez et al. (47)	1	1	1	1	1	1	1	1	1	1	1	0	1	1	1	1	1	1	13
Till (b) et al. (18)	1	1	1	1	1	1	1	1	1	1	1	1	0	0	1	1	1	1	14

#1. In the abstract, an informative and balanced summary of what was done and what was found is provided. #2. Explain the scientiﬁc background and rationale for the investigation being reported. #3. State clear, speciﬁc objectives and/or any pre-speciﬁed hypotheses.#4.Describe the setting, locations, and relevant dates for data collection. This must include information on sport context, type, level of competition, and competition year(s) for data collected to be scored as a “1”. #5a. Give characteristics of study participants (must include age, sex, skill level, overall number and nationality). #5b. Describe the procedure for selecting and grouping athletes in the context under examination (e.g., by birthdate or weight) and how participants were categorised for study purposes (e.g., application of a cut-off date to determine birth quartile). #5c. Describe the source and procedure for obtaining the sample (e.g., obtained from an online roster, provided by a sport governing body). #6. Explain and report the reference baseline distribution (e.g., equal distribution vs. population birth rate). #7a. Clearly describe all statistical methods, including speciﬁc analytical methods used to examine subgroups. #7b. Explain how duplicates (if applicable) and missing data were addressed, or incomplete data were handled. #8. Report the number or percentage of participants found in each quartile/semester (and subcategory if applicable). #9. Provide statistical estimate(s) and precision (e.g., 95% conﬁdence interval) for each sample or subgroup group examined. #10a. *post-hoc* comparisons between quartiles (e.g., Q1 vs. Q4) are provided when appropriate (i.e., overall test is signiﬁcant). #10b. A measure of effect size is provided (e.g., Cramer's V, phi coefﬁcient, Cohen's w). #11. A summary of key results with reference to study objectives is provided. #12. Discusses limitations of the study, taking into account sources of potential bias, confounding factors or imprecision. #13. A cautious overall interpretation of results considering objectives and relevant evidence. #14. Discusses the generalizability of the study results to similar or other contexts.

### 3.4 Meta-analyses

#### 3.4.1 Male players: overall synthesised data

Male rugby players' birth distributions within age-group relative age quartiles were 34.8, 26.1, 22.1, and 16.9%, respectively in Q1, Q2, Q3, and Q4. Overall meta-analysis comparing Q1 and Q4 distributions revealed a significant over-representation of Q1 vs. Q4 players [OR = 1.99 (1.62, 2.43), Z = 6.75, *p* < 0.001]. The *Q* statistic indicated heterogeneity across the samples, suggesting the true effect size differed amongst included studies (*Q* = 540.93, *p* < 0.001). Further, 95.6% [94.4, 96.5] of the variance in the observed effects was attributed to true effects, with a *T^2^* value of 0.21 [0.12, 0.50]. This indicates substantial between-study heterogeneity, and therefore the pooled estimate should be interpreted as an average effect across heterogeneous rugby contexts rather than as a uniform effect applicable to all settings. Figure 2a illustrates a funnel plot based on male studies, revealing potential asymmetry in the Q1 vs. Q4 log Odds Ratios. Specifically, more studies are concentrated on the right-side of the overall effect estimate, with fewer studies below the overall effect estimate and where the standard error is larger. The Egger test confirmed the presence of asymmetry (*t* = 4.74, *p* < 0.001). The follow-up Trim-and-Fill method was applied, yielding an adjusted, but still significant, pooled estimate OR Q1 vs. Q4 of 1.32 [1.02, 1.73] (*n* = 36 imputed samples within studies). This substantial attenuation compared with the unadjusted model suggests that the original pooled male effect may have been inflated by publication bias and/or small-study effects.

#### 3.4.2 Female players: overall synthesised data

In female studies, the age quartile distributions were 27.5, 25.6, 23.6, and 23.6%, respectively in Q1, Q2, Q3, and Q4. Overall, the meta-analysis comparing Q1 vs. Q4 revealed significant but low over-representation of Q1 players [OR = 1.15 (1.03, 1.29)]. The *Q* statistic indicated heterogeneity across samples, suggesting the true effect size differed amongst studies (*Q* = 25.69, *p* < 0.001). Further, 84.4% [65.1, 93.0] of the variance in the observed effects was attributed to true effects, with a *T^2^* value of 0.01 [0.002, 0.07]. As for the male model, this level of heterogeneity indicates that the pooled estimate should be interpreted cautiously and as an average effect across heterogeneous contexts. Figure 2b shows a funnel plot based on female data. The Egger test confirmed no asymmetry was present (*t* = −0.03, *p* = 0.98) based on OR Q1 vs. Q4 study estimates in the five studies available.

#### 3.4.3 Subgroup analyses: youth vs. senior male players

**Figure 3:**
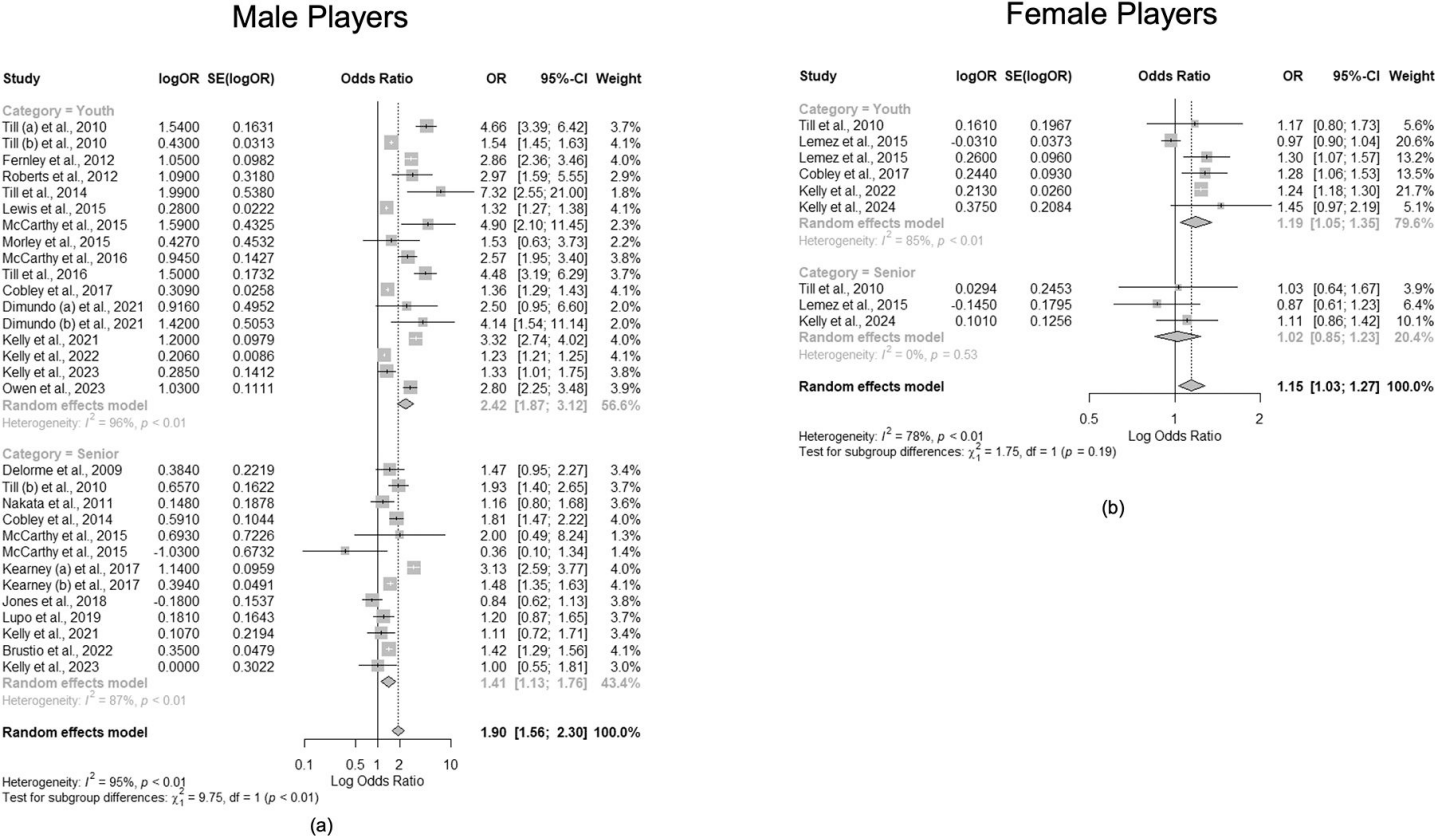
Forest plot summarizing the meta-analysis results for male (**(a)** left panel) and female (**(b)** right panel) players, divided into youth (upper) and senior (lower) categories. The forest plots present Odds Ratio (OR) estimates with 95% confidence intervals (CI) for each study, random-effects models for each subgroup, and overall results from the meta-analysis.

Figure 3a shows the meta-analysis results for the differences between male youth and senior players. The subgroup analysis revealed a significant difference between youth and senior players (*χ*^2^ = 9.75, *p* = 0.002). The *Q* statistic indicated heterogeneity across the samples, suggesting that the true effect size differed among the studies (*Q* = 572.33, *p* < 0.001). Furthermore, 94.9% [93.7, 95.9] of the variance in the observed effects was attributed to true effects, with a T^2^ value of 0.23 [0.14, 0.56]. Therefore, although subgroup differences were examined, these findings should still be interpreted as exploratory in light of the substantial residual heterogeneity. Specifically, the data showed that among young players, the probability of being selected was 2.42 [1.87, 3.12] higher for players born in Q1 than for those born in Q4. In contrast, for senior players, this probability decreased but remained significant [i.e., OR = 1.41 (1.13, 1.76)]. For details about the estimated OR and 95% CI provided by the meta-analysis for each study, see Table 1 (male section).

#### 3.4.4 Subgroup analyses: youth vs. senior female players

Figure 3b reports the meta-analysis results for the differences between female youth and senior players. The subgroup analysis revealed no difference between youth and senior players (*χ*^2^ = 1.75, *p* = 0.18). The Q statistic indicated heterogeneity across the samples, suggesting that the true effect size differed among the studies (*Q* = 35.82, *p* < 0.001). Furthermore, 77.7% [57.6, 88.2] of the variance in the observed effects was attributed to true effects, with a T^2^ value of 0.013 [0.01, 0.07]. As such, subgroup estimates in female samples should also be interpreted cautiously. The data show that among young players, the odds of being selected were 1.19 [1.05, 1.35] higher for players born in Q1 than for those born in Q4. In contrast, for senior players, this probability (though not significant) decreased [OR = 1.02 (0.85, 1.23)]. For details about estimated OR and 95% CI for each study, see Table 1 (Female section).

#### 3.4.5 Subgroup analyses: youth sub-categories in male players

**Figure 4:**
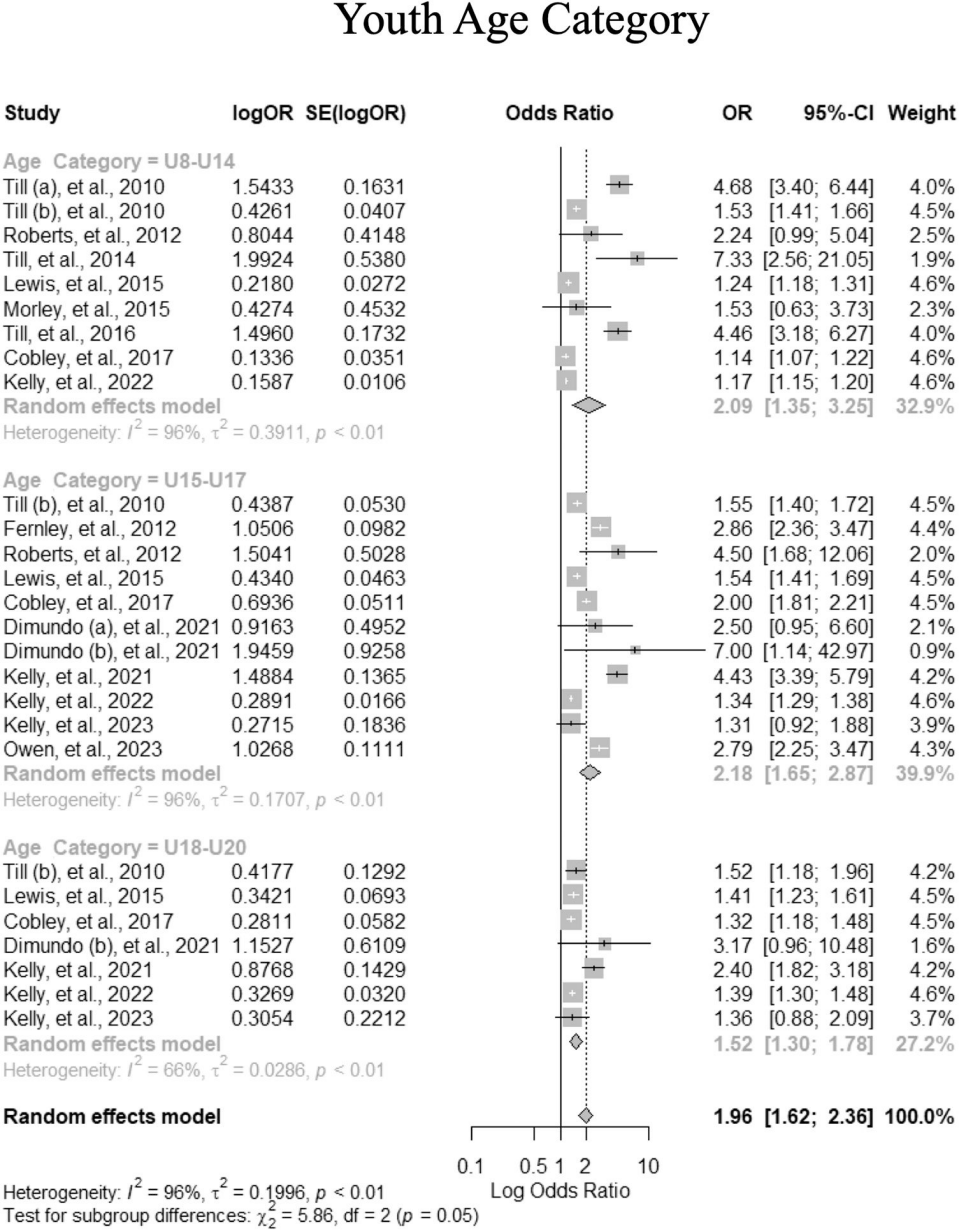
Forest plot summarizing the meta-analysis results for different youth age categories: U8–U14, U15–U17, and U18–U20. The forest plots present Odds Ratio (OR) estimates with 95% confidence intervals (CI) for each study, random-effects models for each subgroup, and overall results from the meta-analysis.

Figure 4 shows the meta-analysis results for differences among the three young age categories (i.e., U7–U14, U15–U17, and U18–U20), while Table 3 shows the study characteristics, quartile distributions (Q1, Q2, Q3, and Q4), and OR between Q1 vs. Q4 according to youth age category. Data showed that the odds of participating is 2.09 [1.35, 3.25], 2.18 [1.65, 2.87], and 1.52 [1.30, 1.78] higher for players born in Q1 than for those born in Q4 for U7–U14, U15–U17, and U18–U20, respectively. Nevertheless, data relevant no significant difference was observed between young age category (*χ*^2^ = 5.86, *p* = 0.053).

**Table 3:** Studies characteristic, quartile distributions and OR between Q1 and Q4 according to youth age category.

**Authors**	**Type**	**N**	**Q1%**	**Q2%**	**Q3%**	**Q4%**	**OR [95% CI]**
Under 8–Under 14							
Cobley, et al. (24)	Rugby League	12,879	27.1	24.1	25.1	23.7	1.14 [1.07, 1.22]
Kelly, et al., (19, 50)	Rugby Union	21,532	28.3	25.6	23.4	22.7	1.24 [1.18, 1.31]
Lewis, et al., (21)	Rugby Union	21,532	28.3	25.6	23.4	22.7	1.24 [1.18, 1.31]
Morley, et al. (42)	Rugby League	84	27.4	21.4	33.3	17.9	1.53 [0.63, 3.73]
Roberts, et al. (44)	Rugby Union	90	42.2	21.1	17.8	18.9	2.24 [0.99, 5.04]
Till (a), et al. (12)	Rugby League	767	45.8	24.9	19.6	9.8	4.68 [3.40, 6.44]
Till (b), et al. (18)	Rugby League	9,688	31.3	26.0	22.2	20.5	1.53 [1.41, 1.66]
Till et al. (45)	Rugby League	81	54.3	27.2	11.1	7.4	7.33 [2.55, 21.05]
Till et al. (46)	Rugby League	650	47.4	23.5	18.5	10.6	4.46 [3.18, 6.27]
Under 15–Under 17							
Cobley et al. (24)	Rugby League	6,327	35.1	26.8	20.5	17.6	2.00 [1.81, 2.21]
Dimundo (a), et al., 2021	Rugby Union	74	33.8	32.4	20.3	13.5	2.50 [0.95, 6.6]
Dimundo (b), et al., 2021	Rugby Union	28	50.0	32.1	10.7	7.1	7.00 [1.14, 42.97]
Fernley, et al. (39)	Rugby Union	1,895	37.6	29.4	19.9	13.1	2.86 [2.36, 3.47]
Kelly et al., (11, 23	Rugby Union	1,114	42.5	27.9	19.9	9.6	4.43 [3.39, 5.79]
Kelly et al., (19, 50)	Rugby Union	58,185	29.1	24.9	24.2	21.8	1.34 [1.29, 1.38]
Kelly et al., (38)	Rugby Union	457	31.3	21.4	23.4	23.9	1.31 [0.92, 1.88]
Lewis et al., (21)	Rugby Union	7,592	30.6	26.1	23.5	19.8	1.54 [1.41, 1.69]
Owen, et al. (34)	Rugby Union	1,424	39.6	26.3	19.9	14.2	2.79 [2.25, 3.47]
Roberts, et al. (44)	Rugby Union	78	46.2	21.8	21.8	10.3	4.50 [1.68, 12.06]
Till (b) et al. (18)	Rugby League	5,733	31.5	25.6	22.6	20.3	1.55 [1.40, 1.72]
Under 18–Under 21							
Cobley et al. (24)	Rugby League	4,737	28.9	25.1	24.2	21.8	1.32 [1.18, 1.48]
Dimundo (b) et al., 2021	Rugby Union	50	38	24	26	12	3.17 [0.96, 10.49]
Kelly et al., (19, 50)	Rugby Union	15,711	29.4	25.9	23.5	21.2	1.39 [1.30, 1.48]
Kelly et al., (38)	Rugby Union	330	28.8	29.1	20.9	21.2	1.36 [0.88, 2.09]
Lewis et al., (21)	Rugby Union	3,361	29.6	25.8	23.6	21	1.41 [1.23, 1.61]
Till (b) et al. (18)	Rugby League	1,011	28.4	26.7	26.2	18.7	1.52 [1.18, 1.96]

#### 3.4.6 Subgroup analyses: competition level in male players

**Figure 5:**
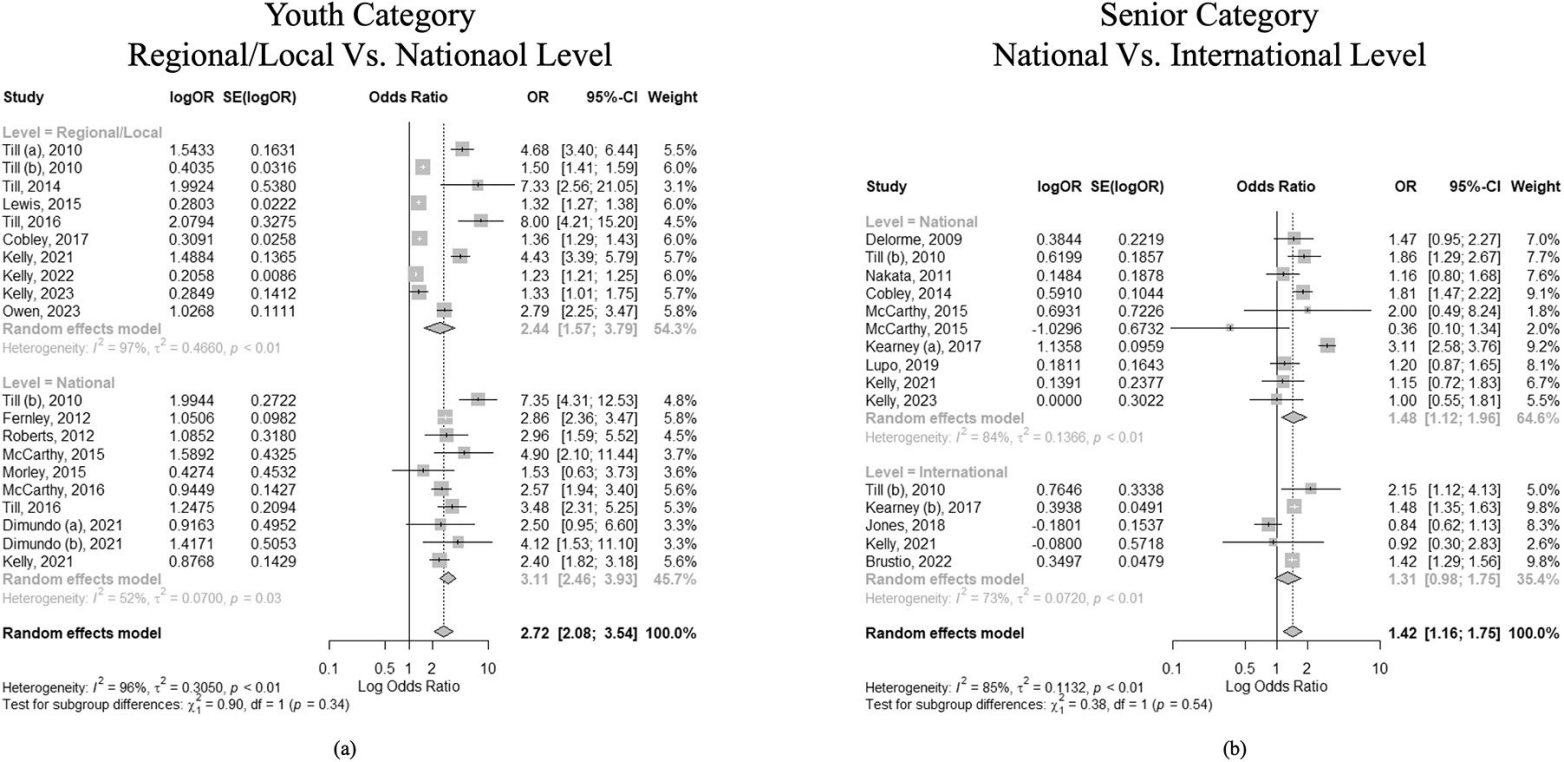
Forest plot summarizing the meta-analysis results for male players, comparing youth (**(a)** left panel) and senior (**(b)** right panel) competition levels. Specifically, the comparisons are as follows: Regional/Local vs. National levels for the Youth Category and National vs. International levels for the Senior Category. The forest plots present Odds Ratio (OR) estimates with 95% confidence intervals (CI) for each study, random-effects models for each subgroup, and overall results from the meta-analysis.

Focusing on the Youth category (pooled data by U7–U14, U15—U17, and U18—U20), there was no significant difference between the Regional/Local and National levels (*χ*^2^ = 0.90, *p* = 0.34). However, the distribution of those born in the Q1 was larger at National level than Regional/Local level. Specifically, the odds of selection for a player born in Q1 were 3.11 [2.46, 3.93] and 2.44 [1.57, 3.79] higher than for a player born in Q4 at National and Regional/Local levels, respectively (Figure 5a). Focusing on the Senior group (Figure 5b), no significant difference was observed between National level and International level (*χ*^2^ = 0.38, *p* = 0.54). However, the odds of being selected as a Q1 player was 1.48 [1.12, 1.96] higher at National level and 1.31 [0.98, 1.74] (not significant) higher at International level relative to a Q4 player.

## 4 Discussion

The primary aim of this systematic review and meta-analysis was to synthesize the prevalence and magnitude of RAEs across RU and RL. The secondary aim was to examine and identify potential moderators of relative age magnitude, including sex, age, and competition level. Principal findings identified a significant overrepresentation of male Q1 (relatively older) players compared to Q4 (relatively younger) players (34.8% vs. 16.9%; OR = 1.99). However, publication-bias analyses indicated that this pooled estimate was likely inflated, as the Trim-and-Fill procedure substantially attenuated the male effect [adjusted OR = 1.32 (1.02, 1.73)]. Accordingly, this result indicated a modest effect than the unadjusted pooled estimate. A smaller effect was observed in female players (Q1 = 27.5% vs. Q4 = 23.6%; OR = 1.15). Age appeared to contribute to differences in overall RAE magnitude, particularly in male youth vs. senior comparisons, whereas competition level showed only descriptive variation and should not be interpreted as a confirmed moderator on the basis of the present subgroup analyses. Among male players, RAEs were more pronounced in the youth category compared to the senior category (OR = 2.42 and OR = 1.41, respectively), whereas in female players, RAEs were only evident in the youth category [OR = 1.19 (1.05, 1.35)] and not the senior category (OR = 1.02). Although not statistically different, RAE magnitude was higher in the younger male youth age categories (U7–U14: OR = 2.09; U15–U17: OR = 2.18) compared to the oldest male youth age category (U18–U20: OR = 1.52), and subsequently increased with competition level, particularly (i.e., Regional/Local level: OR = 2.14; National level: OR = 2.76).

### 4.1 Male players

Overall synthesized data in male players identified a skewed quartile distribution, with Q1 players significantly overrepresented relative to Q4 players. The unadjusted pooled model suggested that Q1 players had approximately twofold greater odds of representation than Q4 players. However, the overall pooled estimate should be interpreted with caution because between-study heterogeneity was substantial, indicating marked variability across rugby contexts. Indeed funnel-plot asymmetry and the Egger test suggested possible small-study and/or publication-related bias, and the subsequent Trim-and-Fill procedure attenuated the pooled estimate. Studies showing no or lower effect sizes with or without sufficient participants sampling may have not been published and thereby were identifiable in systematic searches.

When comparing differences between youth and senior male players, more pronounced RAEs were apparent in youth categories. Findings suggest RAE magnitude decreases during transition from youth to senior levels. Amongst youth players, Q1 players odds of being selected were ∼2.5 times higher than Q4, corresponding to an increase of ∼142% in participation number. In the senior category, the equivalent Q1 odds were ∼1.5 times higher, corresponding to an increase of ∼42% in participation number. These results are consistent with previous RAE in sport meta-analyses (2, 7, 20), with age identified as an RAE moderator. These findings are also consistent with developmental systems approaches to RAEs (9), whereby selection advantages emerge through the interaction of individual, task, and environmental constraints rather than from chronological age alone. Being relatively older during early stages sport participation stages may increase the likelihood of selection (6, 7, 18). Reason identified for this occurrence have often been connected with developmental factors, including anthropometric (e.g., height and weight) (2, 7), cognitive developmental (7, 48), biological maturity (49), and social evaluation processes (e.g., coach evaluation relative to age-group peers) (9). These factors and processes are also purported to influence individual psychology, such as perceived competence. Thus, overall, Q1 players are more likely to outperform, as well as be recognized and rewarded for their investment relative to their younger peers (10–12, 50). This may be particularly evident in youth rugby, where anthropometric and physiological characteristics are highly contributory to performance (10–12). A similar pattern has also been reported in another high-level youth sport context (51). Whilst RAEs magnitudes diminished from age group youth level to senior male players, they remained statistically significant. These findings may potentially reflect residual “knock-on RAEs” from youth stages, as Q1 overrepresentation persisted into senior levels. Previous studies also attributed RAE reductions to an increase in the proportion of Q4 players, who progressively become more integrated (4). The increase and even overrepresentation of relatively younger players in later age/senior competition stages has been referred to as the “underdog hypothesis” (52). More specifically, Q4 players might overcome initial selection obstacles and have a greater likelihood of selection as adults (28, 41, 53). Similar findings have been noted in other sports, such as soccer (4, 54) and track and field sprinters and jumpers (26). The hypothesis proposes relatively younger players, if still retained in a sport system, have in the long-term develop superior technical and psychological skills (e.g., resilience), due to their experiences of challenge and disadvantage in earlier years of involvement (55, 56). Conversely, initial anthropometric and physiological advantages from being relatively older which then dissipate over time may subsequently hinder long-term development, eventually leading to drop out (55, 56). It is worth noting that research on this phenomenon at senior levels is less extensive compared to youth studies, with limited exploration across diverse sociocultural contexts.

Our findings also corroborate with an expanding body of studies indicating RAE magnitudes intensify with competition level. More specifically, the odds ratio between Q1 and Q4 players was approximately 2.7 and 2.4 times higher at the National and Regional/Local level respectively. Albeit no significant differences were apparent between RAE magnitudes at National and Regional levels, a more pronounced trend with higher competition level is indicated. The trend could be attributed to an increased performance emphasis and game demand needs for players with greater anthropometric, physical and cognitive propensities (2, 7). With greater competition for fewer selective places, heightened selection pressure (aka—depth of competition hypothesis) along with a performance-related outcomes may also account for increasing RAE magnitudes (57, 58). Standing in contrast, while RAE were still evident, no further increases in RAE magnitude were apparent from National vs. International selection level (Q1 v Q4 = 1.5 and 1.3 respectively). However, present figures may be affected by the limited number of studies examining international rugby players, and due to conflation with age trends.

### 4.2 Female players

Observed RAE trends in female players were of lesser overall magnitude (OR = 1.15), aligning with prior individual studies and reviews (18, 19). Several factors and/or processes may account for these differences. For instance, the timing of maturational growth spurts maybe one key contributing factor, with females progressing through maturational growth stages earlier relative males (normative age = 12–12.5 v 14–14.5 years), whilst still acknowledging peak growth time points can still vary considerably (e.g., up to 5-years) within the sexes (59). Alongside earlier maturation, there is corresponding greater variability in anthropometric change, with potentially positive and negative influences on performance (60, 61). Thus, relative age influences may dissipate at earlier chronological age time points. Participation number differences, depth of competition and selection dynamics may also relate to lower RAE magnitudes (62). Despite recent growth in female rugby participation, females still comprise only one-quarter of global rugby players (11). More limited player pools within nations may reduce depth of competition and selection, thereby reducing RAE magnitudes observed [56]. The research imbalance on examination of female sports may also be contributing, underestimating true RAE magnitudes apparent. While funnel plot symmetry suggested no publication bias in female samples, RAE-based age differences were only descriptively apparent between youth and senior female players. Overall, several factors and/or process may account for present female-based findings, present RAE estimates may not be representatively valid, and actual RAEs may change over time with expanding game structure (i.e., engagement & competition depth).

### 4.3 Limitations and future directions

A key limitation of the present meta-analysis was the substantial between-study heterogeneity observed across the main models and several subgroup analyses. This indicates that the magnitude of the pooled effects varied considerably across samples and contexts, thereby limiting the interpretability and generalizability of a single summary estimate. Consequently, subgroup analyses should be interpreted as exploratory rather than definitive tests of moderation. In addition, publication-bias analyses suggested that the overall male pooled estimate may have been inflated. The substantial attenuation observed after the Trim-and-Fill procedure indicates that the unadjusted pooled male effect should not be interpreted as a precise estimate of the underlying average magnitude of RAEs in rugby. Rather, it should be viewed as a potentially upwardly biased summary that requires cautious interpretation. This meta-analysis focused on the comparison between Q1 and Q4 as an *a priori* extreme-quartile contrast intended to provide a consistent and directly interpretable summary of relative age differences across heterogeneous rugby samples. This approach allowed the comparison of the relatively oldest and relatively youngest players within the annual selection year using a common effect-size metric across studies. However, this strategy does not fully capture the complete quartile distribution and may overlook non-linear or non-monotonic patterns across Q2 and Q3. Therefore, Q1–Q4 odds ratios should be interpreted as a pragmatic proxy for the relative age gradient rather than as a complete representation of the birth-quartile distribution. This is particularly relevant because Q1 is not always the most represented quartile (13, 40, 43, 47) and patterns such as the so-called “Q2 conundrum” may occur, especially in female samples. Additionally, we compared the observed distribution against an assumed equal distribution of 25% per birth quartile. However, this assumption may not fully reflect true population birth distributions and should therefore be considered a simplifying analytical assumption rather than a universally valid reference distribution. The present study included data from both RU and RL, as these sports share significant similarities in performance profiles and fundamental skills. Findings suggest that RAEs vary based on the reference sample and context within each sport. Specifically, in contexts with a larger number of players, RAEs tend to be more pronounced (7). Given the global distribution and popularity of these two disciplines that differ significantly (i.e., RU being more widespread), there is potential for bias in our analysis due to the combined samples. However, the authors believe that the broader perspective gained by analyzing RAEs across both rugby types outweighs this potential bias. Moreover, it is necessary to point out that the intention was to also include the Rugby Sevens format as well, however, based on our searches, there were no studies that have examined RAEs in this context. Therefore, exploring RAEs in both male and female Rugby Sevens will be an important starting point for this format, particularly as it becomes more popular and professionalized (e.g., Olympic sport). Again, our research suggested also that future research should seek to further examine the effect of RAEs in female sport contexts where minimal data are available. This review only included papers that were published and written in English. This means that national association reports written in other languages were not included. Finally, longitudinal player-level analyses were not available in the present review. Therefore, it remains unclear how RAEs evolve across developmental stages and over time within rugby participation and selection systems. Future longitudinal studies would help clarify these trajectories more directly.

## 5 Conclusion

This systematic review and meta-analysis identified consistent prevalence, magnitudes and moderators of RAEs across both RU and RL codes. Overall findings confirmed skewed quartile distributions across male rugby competitions, with weaker RAE magnitudes in female players. Publication-bias analyses indicated that the unadjusted pooled male estimate was likely inflated, and the Trim-and-Fill procedure suggested a more modest average effect. Age appeared to contribute to variation in RAE magnitude, particularly in male youth vs. senior comparisons, whereas competition level showed descriptive variation but not a confirmed moderating effect in the present subgroup analyses. Findings suggested selection pressures likely amplify RAE-related disparities in higher performance level contexts. Results emphasize the need for rugby organizational systems, coaches and associated practitioners to be cognizant of RAE biases, affecting early and age-group stage of ruby. While relatively older (Q1) players may demonstrate anthropometric and physiological attributes befitting performance in the short-term, these may be transient, with the relatively younger developing more substantially toward the latter stages of maturational growth and where technical capabilities may become more influential. Addressing RAE bias and better considering the transient nature of player trajectories, could all benefit broader participation, player development, as well as player evaluation and selection across rugby codes.

## Data Availability

The original contributions presented in the study are *included* in the article/Supplementary Material, further inquiries can be directed to the corresponding author.
